# ^68^Ga-PSMA-11 PET/CT for Initial Staging of Unfavorable Intermediate-Risk and High-Risk Prostate Cancer Predicts Overall Survival: An IAEA Multicenter Study

**DOI:** 10.2967/jnumed.125.271173

**Published:** 2026-04

**Authors:** Juliano J. Cerci, Stefano Fanti, Enrique E. Lobato, Rakesh Kumar, Jolanta Kunikowska, Akram Al-Ibraheem, Maisarah Nasir, Francisca Redondo Moneda, Osvaldo Garcia, Mohamad Haidar, Fuad Novruzov, Ozlem Kucuk, Umut Elboga, Murilo de Almeida Luz, Diana Paez

**Affiliations:** 1 Quanta Diagnostico e Terapia, Curitiba, Brazil;; 2 IRCCS Azienda Ospedaliero–Universitaria di Bologna, Bologna, Italy;; 3 Division of Human Health, International Atomic Energy Agency, Vienna, Austria;; 4 All India Institute of Medical Sciences, New Delhi, India;; 5 Nuclear Medicine Department, Medical University of Warsaw, Warsaw, Poland;; 6 King Hussein Cancer Center, Amman, Jordan;; 7 Institute Kanser Negara, Putrajaya, Malaysia;; 8 Asistencial Sotero del Río, Santiago Clínica Andes Salud, Puerto Montt, Chile;; 9 Instituto Nacional de Cancerologia, Tlalpan, Mexico;; 10 American University of Beirut Medical Center, Beirut, Lebanon;; 11 Nuclear Medicine Department, National Centre of Oncology, Baku, Azerbaijan;; 12 Ankara University, Ankara, Turkey;; 13 University of Gaziantep, Gaziantep, Turkey; and; 14 Icahn School of Medicine at Mount Sinai, New York, New York

**Keywords:** oncology, prostate cancer, PET/CT, PSMA, initial staging, prognosis

## Abstract

Although multiple studies have demonstrated the accuracy of ^68^Ga-PSMA-11 PET/CT, its ability to predict survival outcomes and treatment response remains unclear. This study assessed the prognostic value of ^68^Ga-PSMA-11 PET/CT in staging unfavorable intermediate- or high-risk prostate cancer (PCa) in patients who are candidates for radical prostatectomy. **Methods:** This prospective multicenter trial supported by the International Atomic Energy Agency enrolled 775 patients across 11 countries with newly diagnosed, unfavorable intermediate- or high-risk PCa. Patients underwent ^68^Ga-PSMA-11 PET/CT, after which their disease was categorized as N0M0 (no involvement of local nodes and no metastases), N1M0 (pelvic lymph node involvement), or NxM1 (distant metastases). These findings were then compared with clinical follow-up data. **Results:** Biochemical recurrence rates were 35.4% (N0M0), 68.2% (N1M0), and 77.2% (NxM1). Two-year event-free survival rates were 56.6%, 43.9%, and 26.0% in patients with N0M0, N1M0, and NxM1 disease, respectively. Two-year overall survival rates were 99.3% in patients with N0M0 disease, 99.2% in those with N1M0 disease, and 86.8% in those with NxM1 disease (*P* < 0.001). ^68^Ga-PSMA-11 PET/CT status was the only significant prognostic factor for survival outcomes. **Conclusion:** ^68^Ga-PSMA-11 PET/CT is a robust and independent prognostic marker in patients with unfavorable intermediate- or high-risk PCa and may help tailor treatments and improve outcomes.

## Visual Abstract

**Figure:**
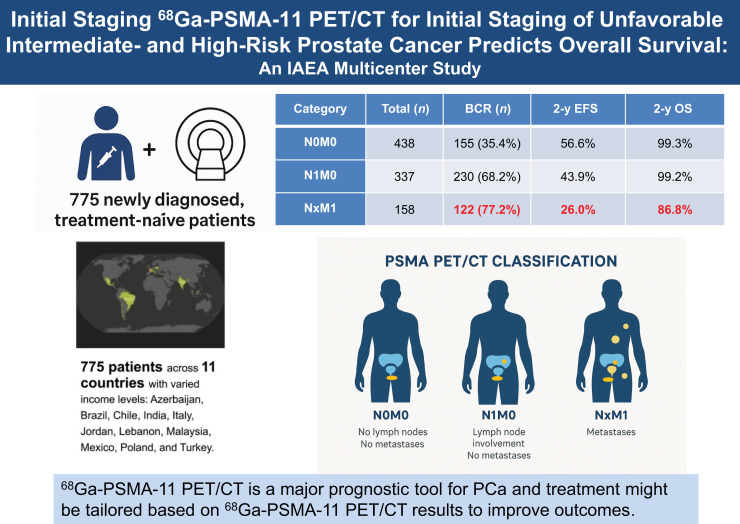

Prostate cancer (PCa) is the second most common malignancy in men and the fifth leading cause of cancer-related deaths worldwide (1). The prognosis of PCa varies widely, depending on the extent of disease at the time of diagnosis. Accurate initial staging is essential for differentiating between patients who may benefit from local therapies (e.g., radical prostatectomy or radiotherapy) and those requiring systemic treatment because of nodal or metastatic disease (2).

Conventional imaging modalities, including MRI, CT, and bone scintigraphy, are routinely used for staging, but their accuracy is limited, particularly in detecting micrometastases and small lymph node involvement. In the past decade, ^68^Ga-labeled prostate-specific membrane antigen (PSMA) PET/CT has emerged as a highly sensitive imaging technique, capable of identifying early metastatic disease that may not be detected by conventional imaging (3–6).

Although multiple studies have demonstrated the superior accuracy of ^68^Ga-PSMA-11 PET/CT, its ability to predict survival outcomes and treatment response remains unclear (7,8).

Determining whether ^68^Ga-PSMA-11 PET/CT can effectively differentiate disease states associated with distinct oncologic outcomes would support its use as a first-line staging tool, enhance treatment decision-making based on imaging findings, and facilitate informed patient counseling regarding the risk and timing of potential treatment failure.

Supported by the International Atomic Energy Agency, this multicenter worldwide trial aimed to evaluate the prognostic impact of ^68^Ga-PSMA-11 PET/CT staging in patients with unfavorable intermediate- or high-risk PCa who were candidates for radical prostatectomy.

## MATERIALS AND METHODS

### Study Design and Population

This prospective, multicenter, international trial enrolled patients with newly diagnosed, biopsy-confirmed, treatment-naïve unfavorable intermediate- or high-risk PCa (9) who were candidates for radical prostatectomy whose disease was staged as nonmetastatic on conventional imaging (MRI, CT, and bone scintigraphy). Patients with evidence of another malignancy, except nonmelanoma skin cancer, were excluded.

This study involved 12 centers from 11 countries, totaling 775 patients: Azerbaijan, 29 patients; Brazil, 180; Chile, 38; India, 160; Italy, 99; Jordan, 64; Lebanon, 30; Malaysia, 43; Mexico, 31; Poland, 69; and Turkey, 32.

Standard forms for data registration were developed and agreed on by the investigators. Data were collected for ^68^Ga-PSMA-11 PET/CT positivity rate, localization of positive findings, and impact on patient management. All centers obtained local ethical clearance for prospective recruitment of patients and data collection, in accordance with national regulations. The institutional review board of each center approved this study, and all patients provided written informed consent.

Studies were performed in accordance with existing procedural international guidelines. On the basis of procedure guidelines for PCa imaging, findings were classified as either positive or negative regarding prostate, seminal vesicle, lymph node, and metastatic involvement (10).

Either analog or digital tomographs were allowed; true whole-body imaging, including lower limbs, contrast-enhanced CT obtained with the application of a diuretic was also allowed. All centers had more than 5 y of experience reporting ^68^Ga-PSMA-11 PET/CT results.

### Imaging and Risk Stratification

All patients underwent ^68^Ga-PSMA-11 PET/CT, and their disease was categorized as N0M0 (no involvement of local nodes and no metastases), N1M0 (pelvic lymph node involvement), or NxM1 (distant metastases).

### Outcomes

The primary outcome was overall survival (OS). Death from any cause was treated as a censoring event. The secondary outcome was event-free survival (EFS), defined as the earliest occurrence of any of the following events: biochemical recurrence (BCR) after radical prostatectomy, defined as a prostate-specific antigen (PSA) level of 0.2 ng/mL or greater confirmed by a second measurement (11); BCR after radiotherapy, defined as an increase in PSA level of at least 2 ng/mL above the nadir, according to the Phoenix definition (12); radiologic progression, defined as local recurrence or the appearance of new metastatic lesions detected on imaging, including PSMA PET/CT (13); or death from any cause.

### Statistical Analyses

A prespecified statistical analysis plan measured time-to-event outcomes from the date of study inclusion, with events recorded at the time of detection. Survival was estimated using Kaplan–Meier curves, with 2-y survival rates and 95% CIs reported. Cox proportional hazards regression models were used to compare the 3 groups in univariate and multivariate analyses unadjusted and adjusted for PSA, Gleason score, International Society of Urological Pathology (ISUP) grade, and age.

Categoric variables are presented as counts and percentages, and continuous variables as means with SDs and medians with ranges. Analyses were conducted using SPSS (IBM) and independently reviewed by a statistician.

## RESULTS

### Outcomes Based on ^68^Ga-PSMA-11 PET/CT Staging

The clinical characteristics of patients are summarized in Table 1. Most patients (71.6%) met the criteria for high-risk PCa. Detailed treatment data and the impact of PSMA PET findings on surgical management have been previously reported (14).

**TABLE 1.** Clinical Characteristics of Patients

Characteristic	Value
Gleason score	
3 + 4	102 (13.2)
3 + 5	7 (0.9)
4 + 3	231 (29.8)
4 + 4	240 (31.0)
4 + 5	123 (15.9)
5 + 3	3 (0.4)
5 + 4	42 (5.4)
5 + 5	26 (3.4)
ISUP grade	
2	73 (9.4)
3	303 (39.1)
4	280 (36.1)
5	192 (24.8)
High-risk PCa	555 (71.6)
Age (y)	67.35
PSA (ng/mL)	15.8

Qualitative data are number and percentage. Continuous data are median.

**FIGURE 1.**
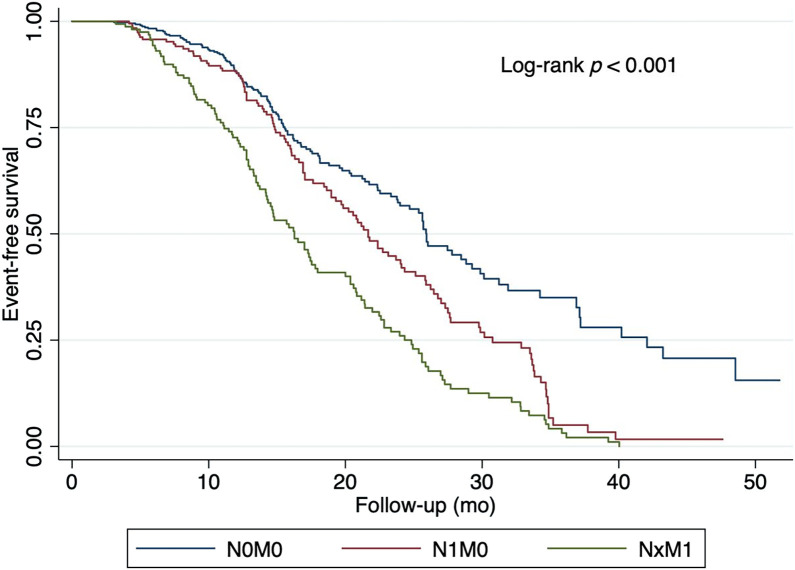
Association between ^68^Ga-PSMA-11 PET/CT and EFS.

**FIGURE 2.**
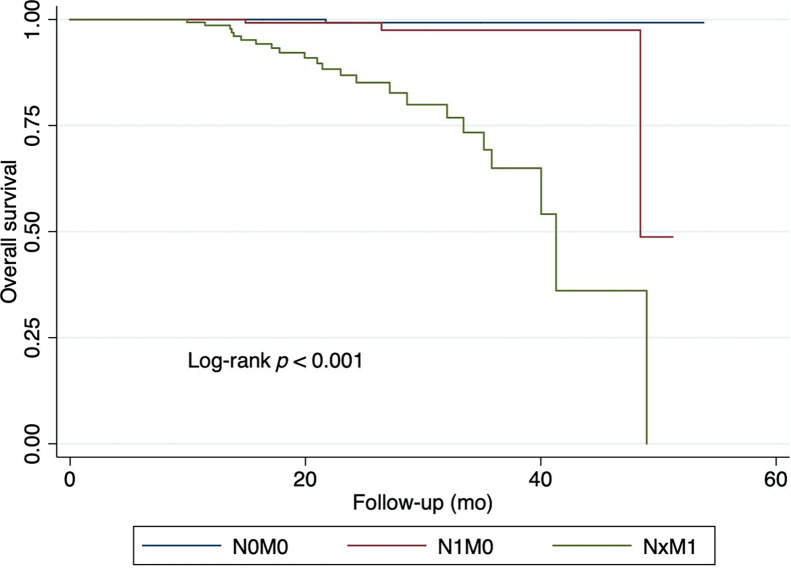
Association between ^68^Ga-PSMA-11 PET/CT and OS.

Survival outcomes differed markedly across PSMA PET–defined staging groups. Patients with N0M0 disease had the highest 2-y EFS and OS rates, whereas those with distant metastases (NxM1) had substantially poorer outcomes (Table 2; Figs. 1 and 2).

**TABLE 2.** Survival Outcomes by Disease Category

				%	
Category	*n*	BCR	No. deaths	2-y EFS	2-y OS
N0M0	438	155 (35.4)	1	56.6	99.3
N1M0	337	230 (68.2)	2	43.9	99.2
NxM1	158	122 (77.2)	23	26.0	86.8

Qualitative data are number and percentage.

### EFS

Univariate analyses found that ^68^Ga-PSMA-11 PET/CT was the only factor significantly associated with treatment failure, with 2-y EFS rates of 56.6% for those with N0M0 disease, 43.9% (hazard ratio [HR], 1.57) for those with N1M0 disease, and 26% (HR, 2.59) for those with metastatic disease (*P* < 0.001) (Table 3).

**TABLE 3.** Results of Univariate and Multivariable Analyses of EFS

Variable	HR	*P* > |*z*|	95% CI
Univariate analysis			
N1M0	1.57	0.00	1.22–2.02
NxM1	2.59	0.00	2.03–3.31
Multivariate analysis			
Disease category			
N1M0	1.59	0.00	1.22–2.06
NxM1	2.65	0.00	2.03–3.46
Age	0.99	0.82	0.98–1.01
ISUP grade	1.15	0.66	0.99–1.33
High-risk status	0.78	0.14	0.57–1.08
PSA level	0.99	0.42	0.99–1.00

In the multivariate analysis, the HR for patients with N1M0 disease was 1.59 (*P* < 0.001), significantly associated with worse EFS rates (Table 3; Fig. 1). Patients with metastatic disease had a HR of 2.65 (*P* < 0.001), which was more strongly associated with poor EFS rates. Age, ISUP grade, high-risk status, and PSA level were not significant predictors of EFS in this model.

### OS

Univariate analyses found that ^68^Ga-PSMA-11 PET/CT was the only factor significantly associated with OS, with 2-y OS rates of 99.3% for patients with N0M0 disease, 99.2% for patients with N1M0 disease, and 86.8% for patients with NxM1 disease (*P* < 0.001) (Table 4). The HR for patients with N1M0 disease was 5.78 (*P* = 0.130), whereas patients with NxM1 disease had a HR of 56.37 (*P* < 0.001).

**TABLE 4.** Results of Univariate and Multivariable Analyses of OS

Variable	HR	*P* > |*z*|	95% CI
Univariate analysis			
N1M0	5.78	0.130	0.59–55.95
NxM1	56.37	0.000	7.54–420.95
Multivariate analysis			
Disease category			
N1M0	4.83	0.176	0.49–47.32
NxM1	50.13	0.000	6.35–395.38
Age	0.99	0.777	0.95–1.03
ISUP grade	1.09	0.758	0.62–1.91
High-risk status	2.81	0.211	0.55–14.19
PSA level	0.99	0.630	0.99–1.00

In the multivariate analysis, patients with N1M0 disease had a HR of 4.83 (*P* = 0.176), which was not associated with worse OS rates. Those with NxM1 disease had a HR of 50.13 (*P* < 0.001), which had a stronger association with poor OS rates (Table 4; Fig. 2). Age, ISUP grade, high-risk status, and PSA were not significant predictors of OS in this model.

## DISCUSSION

To the best of our knowledge, this is the first trial to demonstrate the prognostic value of ^68^Ga-PSMA-11 PET/CT for determining OS as an independent parameter in a large international multicenter cohort of patients with unfavorable intermediate- or high-risk PCa who were candidates for prostatectomy. Our findings confirm that staging of PCa with ^68^Ga-PSMA-11 PET/CT is a strong predictor of treatment outcomes, including OS. Beyond improving staging, ^68^Ga-PSMA-11 PET/CT provides meaningful prognostic information that may guide treatment decisions. Patients classified as having N0M0 disease exhibited significantly better EFS and OS rates compared with those with NxM1 disease, underscoring the prognostic utility of ^68^Ga-PSMA-11 PET/CT.

A few studies have shown that PSMA-defined tumor burden correlates with EFS and OS across various clinical contexts, particularly in patients undergoing salvage radiotherapy after radical prostatectomy. Wenzel et al. demonstrated in more than 1,500 patients that locoregional PSMA-positive findings were highly prognostic for 5-y metastasis-free survival in the setting of salvage radiotherapy in patients with recurrent PCa (HR, 13.8; 95% CI, 7.5–25.2; *P* < 0.001) (15). Emmett et al. (16) similarly reported that PSMA PET/CT was highly predictive of 3-y freedom from progression in men undergoing salvage radiotherapy for BCR of PCa.

In contrast, prognostic data in the initial staging setting remain limited. Hofman et al. (17) evaluated approximately 300 patients with intermediate- to high-risk PCa using baseline ^68^Ga-PSMA-11 PET/CT and found that PSMA-positive nodal disease was associated with a significantly higher risk of treatment failure. Reported EFS rates were 75% for patients with N0M0 disease and 57% for those with N1M0 disease, higher than our findings of 56.6% and 43.9%, respectively. These discrepancies may reflect differences in patient populations. Our cohort consisted exclusively of patients with unfavorable intermediate- or high-risk PCa from a broad international sample, whereas the sample evaluated by Hofman et al. included those with favorable intermediate-risk disease.

Xiang et al. (18) retrospectively evaluated a PSMA PET/CT–derived risk stratification tool in patients with high-risk or very high-risk PCa, categorized as localized and nonlocalized PCa, demonstrating that PSMA PET/CT nomogram classification was significantly associated with long-term oncologic outcomes, such as BCR, development of distant metastases, and PCa-specific mortality. Our results align with these findings but in a wider clinical context, using only PSMA PET–derived disease extent rather than a composite nomogram. PSMA-detected N1M0 and NxM1 disease remained independent predictors of worse EFS rates, even after adjustment for age, ISUP grade, high-risk status, and PSA level. For OS, PSMA-detected metastatic disease was the only significant predictor, with patients with metastatic disease exhibiting a nearly 50-fold higher risk of death.

Sweere et al. (19) also demonstrated the prognostic value of PSMA PET/CT, showing that the clinical tumor stage assessed by PSMA PET was independently associated with BCR-free survival in a multivariate analysis, adjusting for clinical parameters and MRI-derived staging. Karpinski et al. (20) found that combining PSMA PET information with PROMISE criteria improved disease risk classification. While these approaches—such as molecular imaging TNM classification, uptake quantification, and PSMA tumor volume—provide additional refinement, their implementation requires specialized software, standardization, and expertise not widely available. Thus, our use of simple PSMA-based staging categories allows broad clinical applicability across diverse practice settings.

As ^68^Ga-PSMA-11 PET/CT becomes the standard practice for PCa staging, its prognostic implications are expected to increasingly influence treatment paradigms. Incorporating PSMA PET–based risk stratification into treatment planning may support more personalized management, including metastasis-directed therapy and intensified systemic therapy for high-risk patients. Ongoing prospective trials are evaluating whether PSMA PET–guided treatment escalation improves long-term outcomes (8). As evidence expands, PSMA PET/CT is likely to redefine risk-adapted management strategies, further solidifying its role as a fundamental tool in initial PCa staging.

### Clinical Implications

In practical terms, ^68^Ga-PSMA-11 PET/CT clearly differentiates patients with a high likelihood of treatment success (N0M0) from those with markedly poorer prognosis (NxM1) who may benefit from intensified systemic therapy. These results highlight the potential of PSMA PET/CT to guide timely and appropriate treatment selection, particularly for patients with PSMA-detected metastatic disease.

### Limitations

This study had several limitations. First, the absence of a randomized control group limits direct comparison with patients staged using conventional imaging alone. Second, treatment regimens varied across centers, introducing potential heterogeneity in outcomes. Third, although the median follow-up time of 36 mo is informative, longer-term data are needed to fully assess the prognostic impact of ^68^Ga-PSMA-11 PET/CT, particularly for patients with more favorable disease who may experience late events.

## CONCLUSION

^68^Ga-PSMA-11 PET/CT is a robust and independent prognostic marker in patients with unfavorable intermediate- or high-risk PCa who are candidates for radical prostatectomy. These findings support routine integration of ^68^Ga-PSMA PET/CT into initial staging for survival predictions, opening the possibilities to more tailored treatment and potentially better outcomes.

## DISCLOSURE

This research was partially funded by the International Atomic Energy Agency. No other potential conflict of interest relevant to this article was reported.

## Notes

KEY POINTS **QUESTION:** Does ^68^Ga-PSMA-11 PET/CT staging provide prognostic information in patients with unfavorable intermediate- or high-risk PCa who are candidates for radical prostatectomy? **PERTINENT FINDINGS:** In this prospective, International Atomic Energy Agency–supported multicenter cohort study of 775 patients from 11 countries, ^68^Ga-PSMA-11 PET/CT staging stratified patients into groups with significantly different EFS and OS rates. **IMPLICATIONS FOR PATIENT CARE:** Staging with ^68^Ga-PSMA-11 PET/CT enables risk-adapted treatment research by identifying patients with poorer prognoses, supporting more individualized PCa management.
